# Circulating tumor cells detected with a microcavity array predict clinical outcome in hepatocellular carcinoma

**DOI:** 10.1002/cam4.3790

**Published:** 2021-03-06

**Authors:** Kazuto Takahashi, Kazuya Ofuji, Katsushi Hiramatsu, Takuto Nosaka, Tatsushi Naito, Hidetaka Matsuda, Katsuya Endo, Masayuki Higuchi, Masahiro Ohtani, Tomoyuki Nemoto, Yasunari Nakamoto

**Affiliations:** ^1^ Second Department of Internal Medicine Faculty of Medical Sciences University of Fukui Fukui Japan; ^2^ Hitachi Chemical Co., Ltd Tokyo Japan

**Keywords:** albumin, CTC, EpCAM, HCC, microcavity array

## Abstract

The present study aimed to establish a novel isolation strategy for circulating tumor cells (CTCs) using a microcavity array (MCA) system and to evaluate the clinical significance of CTCs in hepatocellular carcinoma (HCC). We examined recovery rates of HCC cell lines spiked into whole blood in MCA assay. Circulating tumor cells were isolated from peripheral blood samples (3 mL) of 7 healthy donors (HD), 14 patients with liver cirrhosis (LC), and 31 patients with HCC using the MCA system. Additionally, we investigated the mRNA expression of liver‐specific genes in isolated CTCs using qPCR. The recovery rates were 65.1% (HepG2), 76.7% (HuH7), and 99.0% (PLC/PRF/5). In HD and patients with LC and HCC, the CTC positivity rate (CTCs ≥10) and average CTC number were as follows: HD 0% and 0.1, LC 14.3% and 5.3, HCC 54.8% and 47.6, respectively. The CTC positivity rate in HCC was significantly higher than that in LC (*p* < 0.05). The number of CTCs was significantly higher in metastatic HCC (102.2 ± 160.6) than in localized HCC (8.2 ± 7.7) (*p* < 0.05). The expression of *AFP*, *glypican*‐*3*, *EpCAM*, and *albumin* (*ALB)* genes was detected in isolated CTCs. The positive CTCs (CTCs ≥10) significantly reduced the cumulative survival in patients with HCC (*p* = 0.025), especially in localized patients with HCC (*p* = 0.046). The newly developed MCA system has the potential to isolate CTCs from HCC with high sensitivity, and mRNA expression could be measured from CTCs. Identification of positive CTCs can help predict clinical outcome of patients with HCC. Thus, analysis of CTCs in patients with HCC may provide important information as a novel biomarker in disease progression.

## Graphical abstract

This study describes the detection of circulating tumor cells (CTCs) in the peripheral blood of hepatocellular carcinoma (HCC) patients using a newly developed microcavity array. Our results show that a large number of CTCs was detected in metastatic HCC patients, and mRNA expression of AFP, glypican‐3, EpCAM and albumin was detected in detected CTCs. Identification of positive CTCs or ALB mRNA expression can help predict poor prognosis of HCC patients.

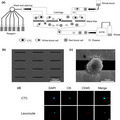

**Abbreviations**
AFP: alpha‐fetoprotein
ALB: albumin
ALT: alanine aminotransferase
CK: cytokeratin
CTCs: circulating tumor cells
DMEM: Dulbecco's modified Eagle medium
EDTA: ethylenediaminetetraacetic acid
EpCAM: epithelial cell adhesion molecule
FBS: fetal bovine serum
GPC3: glypican‐3
HCC: hepatocellular carcinoma
IRB: institutional review board
MCA: microcavity array
NGS: next generation sequencing
PT: prothrombin time
qPCR: quantitative reverse transcription‐PCR
ROC: receiver operating characteristic
SEM: scanning electron microscope

## 1 INTRODUCTION

Liver cancer, including hepatocellular carcinoma (HCC), is currently the second leading cause of cancer mortality worldwide and has an incidence of about 850,000 new cases per year.^1^ HCC represents about 90% of all cases of primary liver cancer.^1^ HCC is complicated by chronic liver disease caused by hepatitis B and hepatitis C predominantly, although the prevalence of obesity and alcohol excess have a major impact, with HCC‐related mortality increasing, especially in elderly patients with metabolic risk factors.^2^ Despite amelioration in diagnostics and clinical treatment, the prognosis of HCC remains poor owing to a high frequency of metastasis and recurrence.^3^ In early‐stage HCC, thermal ablation, surgical resection, or liver transplantation have a potential for radical cure, with a 5‐year survival rate of 50–80% after these therapies.^1^ Once the cancer metastasizes within or outside the liver, treatment options are limited, with the 5‐year survival rate falling below 15%.^4^ Whereas early detection of HCC enhances the success of curative treatment, screening and diagnosis in cirrhotic patients is limited because of the low serum levels of the primary biomarker, alpha fetoprotein (AFP).^4^

As hematogenous expansion is the main route of metastasis and recurrence in HCC,^5^ detection of circulating tumor cells (CTCs) has important clinical significance in patients with HCC. Enumeration and analysis of CTCs, which is called ‘liquid biopsy’ may enable noninvasive monitoring of progression, recurrence, and therapeutic efficiency in cancers, as well as early detection of localized cancers before they metastasize and make curative treatment difficult. CellSearch, a CTC isolation system based on EpCAM antibody‐coated magnetic beads, has been approved by the U.S. Food and Drug Administration for detection of CTCs in breast cancer, colon cancer, and prostate cancer.^6^ Epithelial cell adhesion molecule (EpCAM) is specifically expressed on the surface of epithelial cells and epithelial‐derived tumor cells.^7^ It has been reported that high positivity of CTCs indicates poor prognosis in patients with HCC and is associated with poor clinicopathological parameters, but the characteristically low EpCAM expression in this cancer has made standard CTC measurements difficult.^8^, ^9^ Despite HCC being an epithelial tumor, EpCAM is expressed in only about 35% of HCC cases.^10^, ^11^ Therefore, it may not be appropriate to detect CTCs with the CellSearch system in patients with HCC.

These limitations may be overcome using a recently developed microcavity array (MCA), a novel automated CTC isolation system, which does not rely on EpCAM expression. The MCA system can capture CTCs on a precisely fabricated filter with micro pores, based on the difference in size and deformability between tumor cells and normal blood cells, and cells of size 8 μm or less are removed as they pass through the pores. The MCA system has been reported to have practical clinical application in patients with lung cancer.^12^, ^13^ The aims of this study were to develop a novel CTC isolation strategy using an MCA system and to examine the clinical significance of CTCs in patients with HCC. The primary study was presented at The Liver Meeting ^Ⓡ^ 2018 in San Francisco, CA.^14^

## 2 MATERIALS AND METHODS

### 2.1 Microcavity array (MCA) system

**FIGURE 1:**
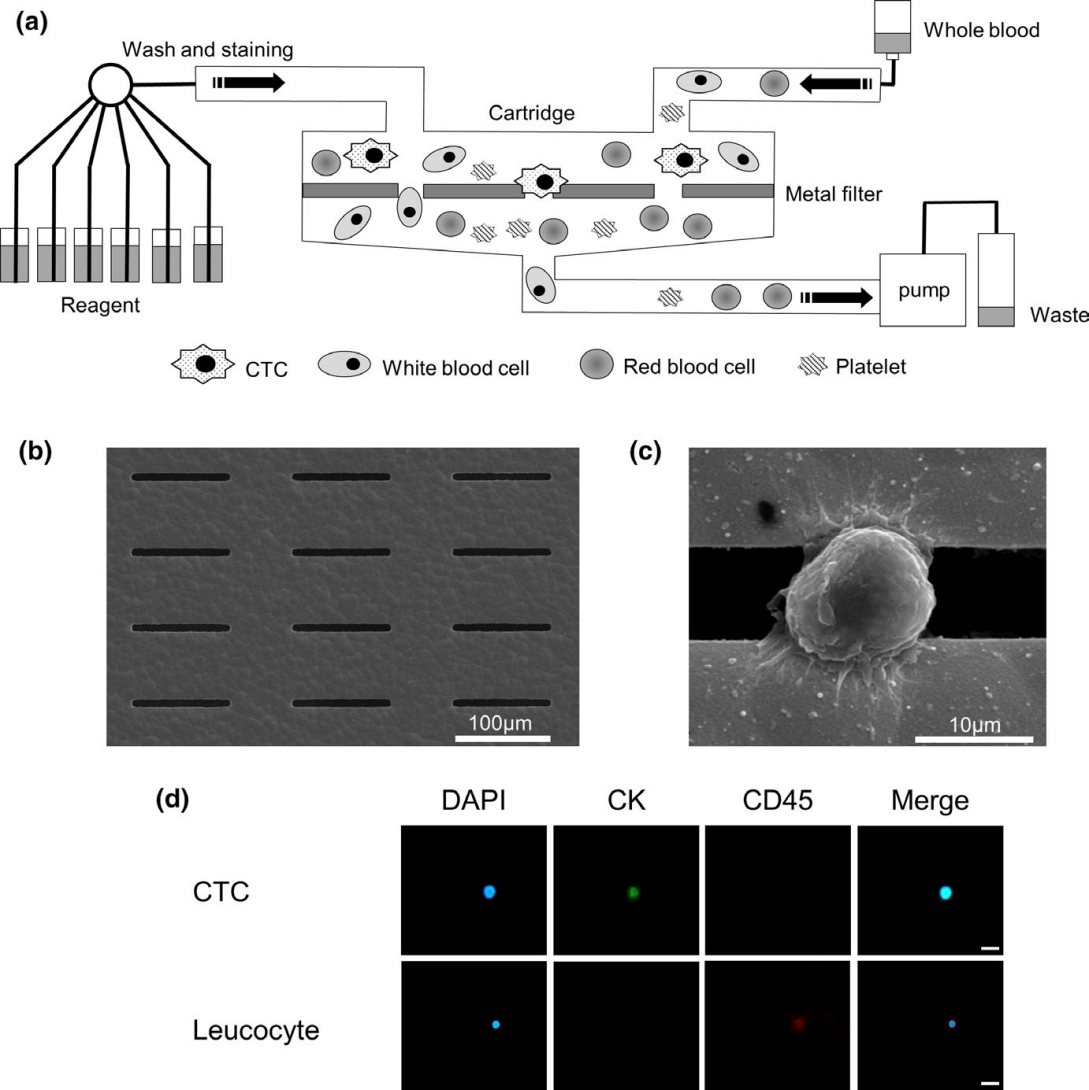
Microcavity array (MCA) system for size‐based isolation of circulating tumor cells. A, Schematic image of the CTC‐enrichment device. B, Scanning electron microscope (SEM) image of the fabricated filter. Scale bar: 100 µm. C, SEM image of a HepG2 cell trapped on the filter. Scale bar: 10 µm. D, A CTC and a leucocyte trapped on the MCA were stained with DAPI and fluorescent‐labeled antibodies that target CK and CD45. Scale bar: 20 µm

To detect the presence of CTCs in whole blood, a filter with a rectangular MCA, which was precisely manufactured in almost the same way as previously reported,^13^ was integrated with a miniaturized device. The MCA system consisted of a blood reservoir, filter‐included cartridge, and individual tubes (Figure 1A). It used a filtration manner with metal filters made of nickel and gold via electroformation. Each of the approximately 3,000 cavities on the filter was fabricated to have a rectangular pore, exactly 8 μm wide and 100 μm long. The form and porosity of the pore were optimized to efficiently capture tumor cells on the filters under low flow resistance condition and let other blood cells pass through by the differences in size and deformability between tumor cells and normal blood cells. Figure 1B shows the scanning electron microscope (SEM) image of the filter, Figure 1C presents an SEM image of a HepG2 cell, which is an HCC cell line, trapped on the filter. The filtration cartridge is connected to one outlet with a peristaltic pump to induce the injection of whole blood and reagents into the cartridge.

### 2.2 Patients and blood samples

Human peripheral blood samples were collected from 7 healthy donors (HD), 14 patients with liver cirrhosis without any cancers (LC), 18 patients with localized HCC (localized), and 13 patients with metastatic HCC (metastatic). The clinical baseline characteristics of healthy donors and patients are presented in Table 1. LC and HCC were diagnosed based on clinical data and computed tomographic signs of LC or HCC. Patients with HCC were classified as localized and metastatic groups according to the presence of metastasis and vascular invasion. The measurement of CTC number started in April 2015. In addition to the measurement of CTC number, mRNA for the qPCR analysis was extracted in consecutive cases from January 2016. The blood samples for the qPCR experiment were collected from 13 patients with localized or metastatic HCC.

**TABLE 1:** Clinical baseline characteristics of healthy donors and patients

Male/Female, n	HD	LC	Localized	Metastatic
	n = 7	n = 14	n = 18	n = 13
	6/1	8/6	16/2	9/4
Age, years	33.0 ± 4.3	70.6 ± 10.4	72.6 ± 10.5	67.0 ± 16.2
Platelets, ×10^4^/µL	28.5 ± 3.7	11.1 ± 6.0	12.8 ± 5.2	15.7 ± 8.6
ALT, U/mL	14.3 ± 3.5	24.9 ± 12.7	28.1 ± 15.3	35.8 ± 20.5
Total Bilirubin, mg/dL		1.55 ± 1.24	0.96 ± 0.63	1.10 ± 0.91
Albumin, g/dl		3.26 ± 0.44	3.52 ± 0.47	3.40 ± 0.52
PT, %		70.8 ± 23.4	78.1 ± 22.6	78.8 ± 9.8
Etiology, n				
HBV/HCV/Alcohol/Others		0/3/6/5	3/8/5/2	4/5/2/2
Child‐Pugh class, n				
A/B/C		4/6/4	11/7/0	9/3/1
AFP, ng/mL		4.8 ± 2.2	210.4 ± 842.1	42716.7 ± 68304.4
Tumor size, mm			27.8 ± 22.0	49.8 ± 27.9
Tumor number, n			2.4 ± 1.8	7.6 ± 3.2

Abbreviation: ALT, alanine aminotransferase; AFP, alpha‐fetoprotein; HD, healthy donor; LC, liver cirrhosis patients without any cancers; Localized, localized HCC patients; Metastatic, metastatic HCC patients; PT, prothrombin time.

Data of age, platelets, ALT, Total Bilirubin, Albumin, PT, AFP, tumor size, and tumor number are presented as mean ±standard deviation.

The whole blood was sampled in 3‐mL EDTA‐containing blood collection tubes (Thermo corporation, Tokyo, Japan) to prevent coagulation, subsequently applied to the MCA system within 5 h at room temperature (15 to 25 ℃). All blood samples were provided in our hospital, and written informed consent from all donors was obtained. This study was approved by the institutional review board (IRB) of our facility (IRB approved number**:** 20140155) and was performed in accordance with the ethical standards stipulated in the 1964 Declaration of Helsinki and its later amendments.

### 2.3 Cell culture

The human hepatocellular carcinoma cell line HepG2 was purchased from the American Type Culture Collection (Manassas, VA, USA), HuH7 and PLC/PRF/5 (Alexander cells) were purchased from the JCRB cell bank (Osaka, Japan). HepG2 cells were cultured in RPMI‐1640 (Thermo Fisher Scientific, Waltham, MA, USA) containing 10% fetal bovine serum (FBS; Thermo Fisher Scientific, Waltham, MA, USA). HuH7 and PLC/PRF/5 cells were cultured in Dulbecco's Modified Eagle Medium (DMEM; Thermo Fisher Scientific, Waltham, MA, USA) containing 10% fetal bovine serum. The cell lines were cultured under their recommended culturing condition.

### 2.4 Cell spiking experiment

The cell lines were cultured for about 14 days and harvested with 0.25% trypsin/ethylenediaminetetraacetic acid (EDTA) (Wako Pure Chemical Industries, Ltd., Osaka, Japan) solution at 37 ℃ before the cell spiking experiment. Tumor cells were counted and spiked into whole blood (3 mL) from a healthy volunteer after they were passed through a cell strainer (Falcon, Flanklin Lakes, NJ, USA) to make a uniform single cell suspension.

### 2.5 Tumor cell capturing and staining

Blood samples containing tumor cells were poured to the reservoir of the MCA system, and then filtrated by using a metal filter through negative pressure via a peristaltic pump. The six regents were injected into the cartridge of the MCA system, Regent 1 was wash buffer for washing cells, and Reagents 2–6 were DAPI, FITC‐cytokeratin antibody, and Alexa594‐CD45 antibody for fluorescent staining. In addition, cell fixation, cell staining, and permeabilization solutions were used. These solutions were sequentially injected into the cartridge after washing.^13^ The isolated cells on the filter were stained immunochemically with cytokeratin (pan‐cytokeratin), DAPI, and CD45. All reagents were provided by Hitachi Chemical Co., Ltd. (Tokyo, Japan).

### 2.6 Identification and enumeration of CTCs

The images of cells on the filter were obtained using the AX80 fluorescence microscope (Olympus Corporation, Tokyo, Japan) and image software cellSens Standard (Olympus Corporation, Tokyo, Japan). The images (at 40× total magnification) were carefully examined. We identified potential CTCs by immunofluorescence staining, cell size, cell shape, and nucleus shape. Cells of size 8 μm or less were not observed in the images because they were removed through the filter pores. Round cells were selected, and irregularly shaped cells were excluded. Furthermore, cells with extremely large nuclei and small punctate nuclei were excluded. Using immunofluorescence staining, we identified cells that were positive for cytokeratin (CK) and DAPI, and negative for CD45 as probable CTCs and those positive for CD45 as contaminating normal leucocytes (Figure 1D). SEM images were obtained using JSM‐6390 (JEOL Co., Ltd., Tokyo, Japan).

### 2.7 Quantitative reverse transcription‐PCR (qPCR)

The RNeasy Mini Kit (Qiagen, Hilden, Germany) was used to extract mRNA from the isolated cells. Subsequently, cDNA was synthesized from RNA with the High Capacity cDNA Reverse Transcription Kit (Applied Biosystems, Foster City, CA, USA), and qPCR was performed with TaqMan Gene Expression Assay and StepOnePlus (Applied Biosystems, Foster City, CA, USA) following preamplification reactions of the cDNA using TaqMan^Ⓡ^ PreAmp Master Mix (Applied Biosystems, Foster City, CA, USA). With reference to previous reports, we selected liver‐specific transcripts that are uniquely expressed in HCC cells, but substantially absent in blood components. AFP and glypican‐3 (GPC3) are well‐known tumor markers. Epithelial cell adhesion molecule (EpCAM) is an epithelial cell‐specific adhesion molecule as mentioned previously.^7^ *Albumin* (*ALB*) is a liver‐specific transcript and one of the most highly expressed genes in HCC, its expression being absent in hematopoietic cells and other normal tissues.^15^, ^16^ The primers and TaqMan^Ⓡ^ probes of *AFP*, *GPC3*, *EpCAM*, and *ALB* were obtained from predesigned assays of Applied Biosystems. The following normalization method was used for the qRT‐PCR analysis. We prepared a calibration curve for liver‐specific gene expression using serially diluted mRNA samples extracted from HCC cell line HuH7 known to express liver‐specific genes. The expression level of liver‐specific genes at the mRNA concentration of 10^−5 ^ng/μL was used as the standard value, and CTC‐derived *AFP*, *GPC3*, *EpCAM*, and *ALB* expression levels were shown as relative ratios to the standard value.

### 2.8 Statistical analysis

Statistical analysis was performed with SPSS Statistics Version 25 software (SPSS Inc., Chicago, IL, USA). Differences were considered statistically significant between two or among multiple groups at a *P*‐value of 0.05 or less (Chi‐squared test, Mann–Whitney *U* test, and Tukey's multiple comparison test). Receiver operating characteristic (ROC) analysis confirmed the optimal cut‐off based on the Youden index. Cumulative survival rate was assessed with the Kaplan–Meier method, and comparison of survival rates among groups was performed with the log‐rank test.

## 3 RESULTS

### 3.1 Recovery rates of HCC cell lines using the MCA system

**FIGURE 2:**
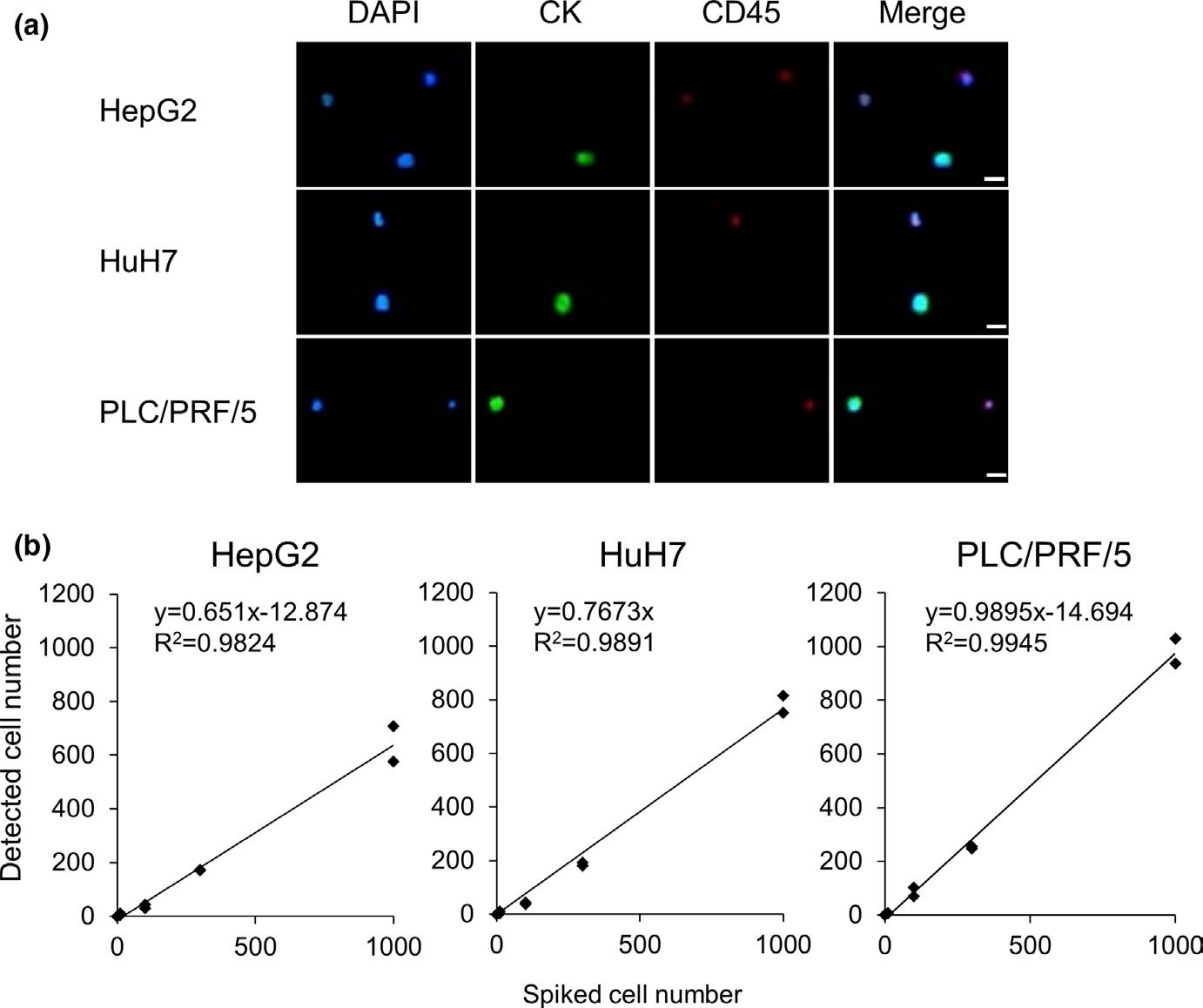
A, HCC cell lines (HepG2, HuH7, and PLC/PRF/5) trapped on the MCA were stained with DAPI and fluorescent‐labeled antibodies that target CK and CD45. Scale bar: 20 µm. B, Recovery rate of HepG2, HuH7, and PLC/PRF/5 cells. HCC cells (0, 10, 100, 300, and 1,000 cells) were spiked into 3 mL of whole blood collected from a healthy volunteer. The number of cells spiked (x‐axis) is plotted versus the number of cells detected (y‐axis)

To verify the MCA system performance, the recovery rate of tumor cells was investigated using HCC cell lines (HepG2, HuH7, and PLC/PRF/5). HCC single cells (0, 10, 100, 300, and 1000 cells) were spiked into 3 mL of whole blood collected from a healthy volunteer and then processed by a MCA assay. The analysis was run in duplicates. Fluorescence microscope images of the HCC cells recovered on the filter were obtained (Figure 2A). Those images demonstrated tumor cells characterized by a pattern of immunofluorescence such as positive for CK and DAPI, and negative for CD45. Some cells characterized by another pattern of immunofluorescence, considered as leukocytes were also captured on the filter. These individual cells were easily distinguished from each other because they were accurately and individually trapped on the aligned arrays of filters, as well as the difference in their characteristics of immunofluorescence.

Figure 2B shows the recovery rates of HepG2, HuH7, and PLC/PRF/5 cells. The spiked number of these cells was plotted against the number of cells detected. Linear regression of the number of detected tumor cells versus the number of spiked tumor cells yielded a slope, an intercept, and a correlation coefficient (R^2^). The average recovery rates of HepG2, HuH7, and PLC/PRF/5 cells were 65.1%, 76.7%, and 99.0%, respectively. No tumor cells were detected in the samples that did not spike with any tumor cells. The results suggested that the MCA system was capable of isolating tumor cells sensitively from whole blood with high recovery rates.

### 3.2 Relative mRNA expression of recovered HCC cell lines

**FIGURE 3:**
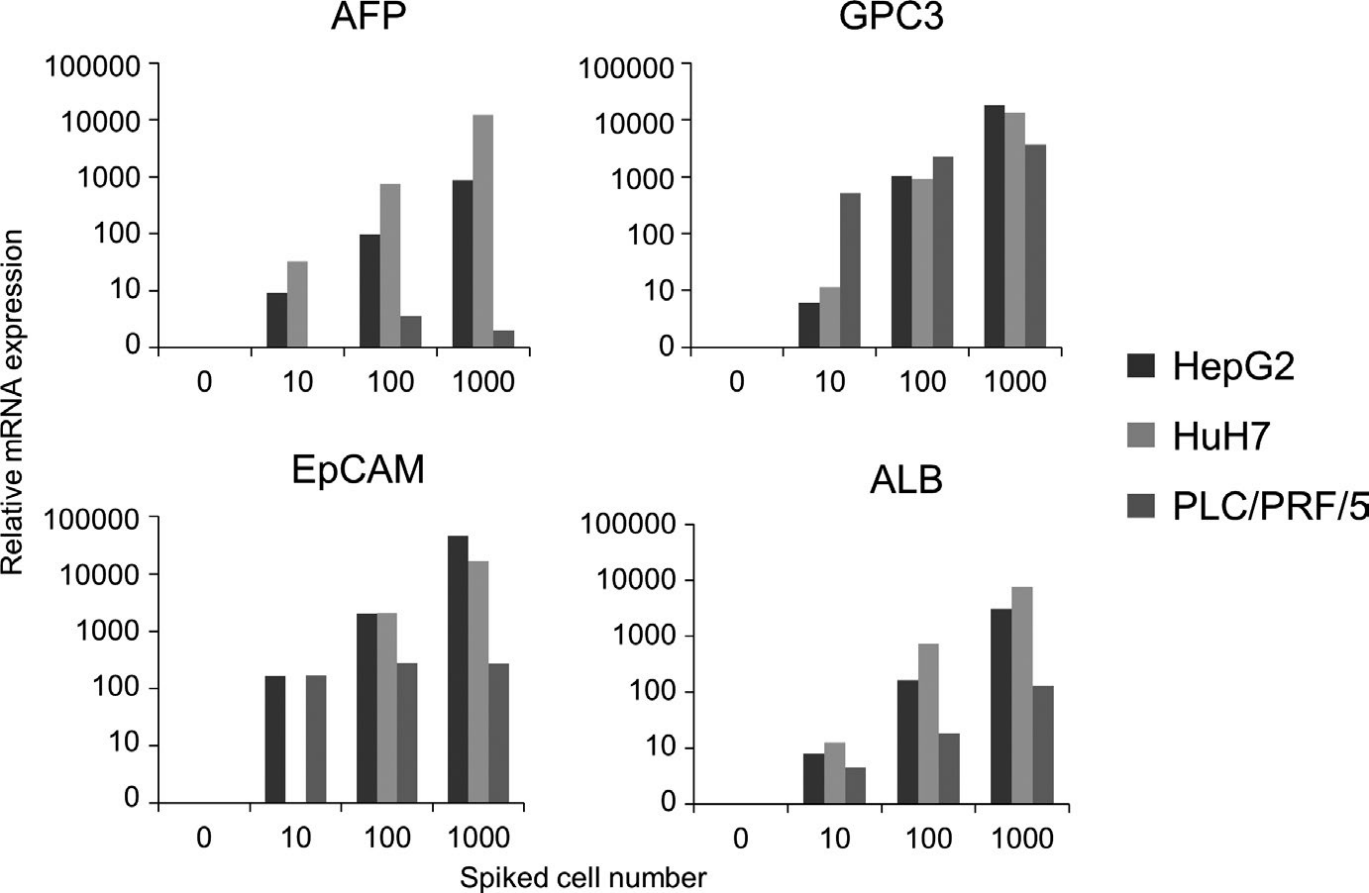
Relative mRNA expression of liver‐specific genes from HCC cells (HepG2, HuH7, and PLC/PRF/5) was analyzed by qPCR. HCC cells were spiked into 3 mL of whole blood and recovered using the MCA system. The number of cells spiked (x‐axis) is plotted versus relative mRNA expression (y‐axis)

To detect liver‐specific gene expression from recovered HCC cell lines, extracted mRNA was examined using qPCR. The data were presented in terms of relative expression to that of 10^−5 ^ng/μL HuH7 mRNA as a positive control. The four liver‐specific genes, that is, *AFP*, *GPC3*, *EpCAM*, and *ALB*, were detected in all HCC cell lines. Furthermore, the levels of relative mRNA expression were found to increase with increasing number of spiked cells (Figure 3). We confirmed that relative mRNA expression from minimum 10 HCC cells spiked into whole blood was detectable by qPCR by using this technology.

### 3.3 Detection and quantitation of CTCs in clinical samples

**FIGURE 4:**
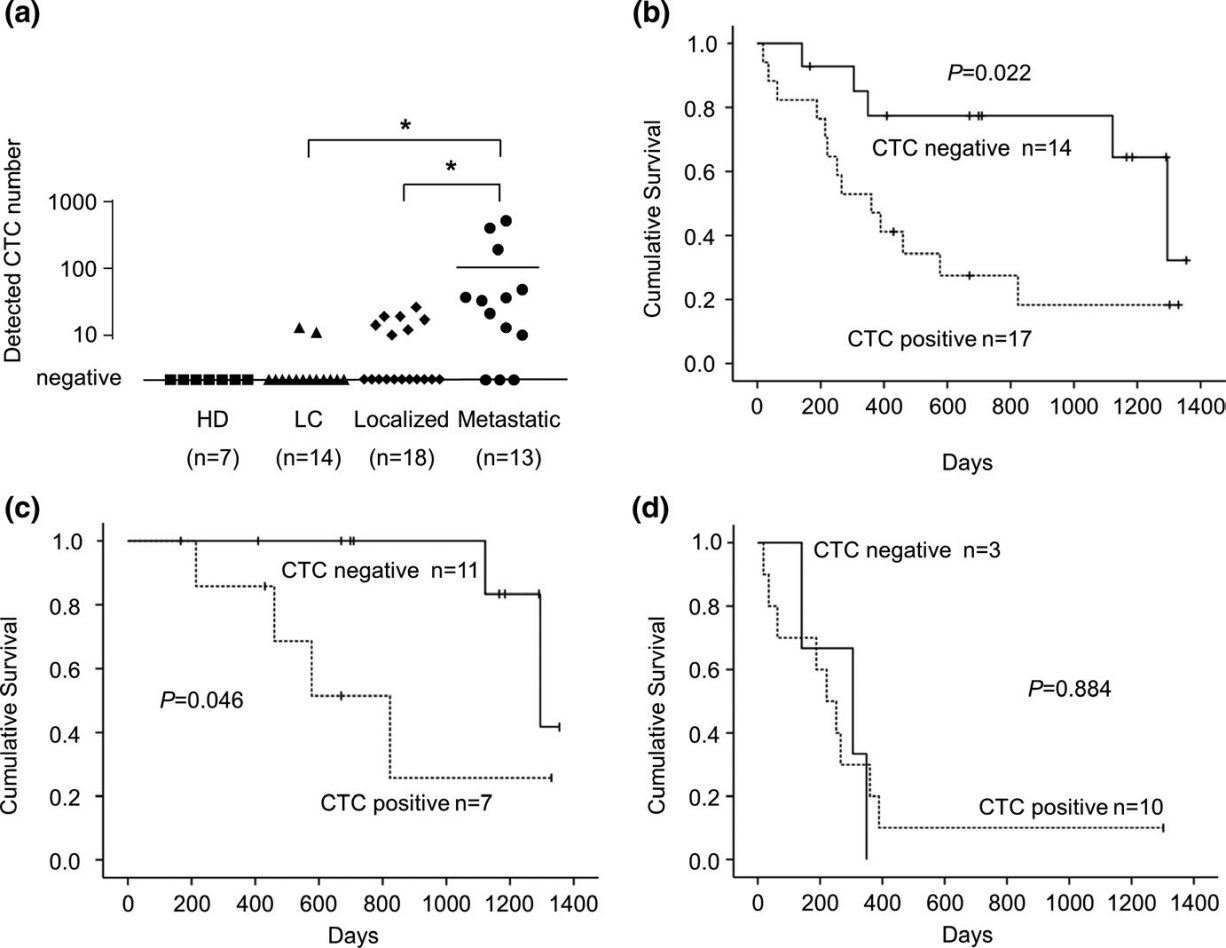
A, Isolated CTC numbers in 3 mL of whole blood from patients using the MCA system, including 7 healthy donors (HD), 14 patients with liver cirrhosis (LC), 18 patients with localized HCC (localized), and 13 patients with metastatic HCC (metastatic). We defined the cut‐off of a CTC number as 10 from the result of the ROC analysis. *p < 0.05. B, The Kaplan–Meier analysis of cumulative survival in patients with HCC with positive or negative CTCs. C, The Kaplan–Meier analysis of cumulative survival in patients with localized HCC with positive or negative CTCs. D, The Kaplan–Meier analysis of cumulative survival in patients with metastatic HCC with positive or negative CTCs

To detect CTCs in the peripheral blood of patients using the MCA system, we quantitated CTC numbers in 3 mL of whole blood collected from the patients (LC, localized, and metastatic groups) and the healthy donor (HD). Additionally, to exclude false positives for CTCs, we performed ROC analysis based on the CTC numbers in patients and healthy donors (Fig. S1). We defined the positive cut‐off of CTC numbers for definite HCC diagnosis as 10. Furthermore, we divided the patients into CTC‐positive (CTC ≥10) and CTC‐negative (CTC <10) groups in this study. A comparison of clinical characteristics between the two groups revealed that AFP and tumor size were significantly different between the groups, but not other characteristics (Table 2). In HD and patients with LC and HCC, the CTC positivity rate (CTCs ≥10) and average CTC number were as follows: HD 0% and 0.1, LC 14.3% and 5.3, HCC 54.8% and 47.6, respectively. The CTC positivity rate in HCC was significantly higher than that in LC (*p* < 0.05). The CTC positivity rate in the localized and metastatic HCC groups was 38.9% (7/18) and 76.9% (10/13), respectively. The mean number of detected CTCs in the LC and localized and metastatic HCC groups was 5.3, 8.2, and 102.2, respectively, and there were significant differences between the metastatic group and other groups (*p* < 0.05, Figure 4A).

**TABLE 2:** Clinical characteristics in CTC‐positive and CTC‐negative HCC patients

Male/Female, n	CTC‐positive	CTC‐negative	*p* value
	n = 17	n = 14	
	13/4	12/2	0.664
Age, years	72.0 ± 12.4	68.1 ± 14.2	0.493
Platelets, ×10^4^/µL	15.4 ± 8.1	12.4 ± 4.8	0.493
ALT, U/mL	30.9 ± 19.0	31.9 ± 16.9	0.739
Total Bilirubin, mg/dL	1.02 ± 0.69	1.02 ± 0.82	0.830
Albumin, g/dL	3.49 ± 0.54	3.46 ± 0.46	0.922
PT, %	74.0 ± 23.2	82.0 ± 12.1	0.262
Etiology, n			
HBV/HCV/Alcohol/Others	4/7/2/4	3/6/5/0	0.490
Child‐Pugh class, n			
A/B/C	11/5/1	9/5/0	0.632
AFP, ng/mL	32283.2 ± 62597.6	735.1 ± 1841.4	0.029
Tumor size, mm	46.9 ± 30.0	25.0 ± 15.8	0.036
Tumor number, n	5.5 ± 3.6	3.5 ± 3.2	0.109

Abbreviation: ALT, alanine aminotransferase; AFP, alpha fetoprotein; PT, prothrombin time.

Data of age, platelets, ALT, Total Bilirubin, Albumin, PT, AFP, tumor size, and tumor number are presented as mean ±standard deviation.

To evaluate the clinical implication of CTCs, we assessed the cumulative survival rate in patients with HCC by Kaplan–Meier method. In terms of the prognosis, 18 patients in our cohort of 31 patients with HCC (58.1%) died within the observation period with a median time of death of 577 days. The cumulative survival rate for CTC‐positive group was significantly lower than that for CTC‐negative group (median, 360 days versus 1295 days; *p* = 0.022) (Figure 4B). In addition, we assessed the cumulative survival of patients in the localized and metastatic HCC groups. For localized HCC, the cumulative survival rate of patients in the CTC‐positive group was significantly lower than that of patients in the CTC‐negative group (median, 823 versus 1295 days; *p* = 0.046) (Figure 4C). For metastatic HCC, there was no difference between the CTC‐positive and CTC‐negative groups (median, 221 versus 305 days; *p* = 0.884) (Figure 4D).

These results suggested that a large number of CTCs may be circulating in the peripheral blood of advanced HCC patients complicated with extrahepatic metastasis. Furthermore, the detection of 10 or more CTCs with the MCA system predicted poorer prognosis in HCC patients, especially in early‐stage HCC patients.

### 3.4 Molecular characteristics of the detected CTCs in clinical samples

**FIGURE 5:**
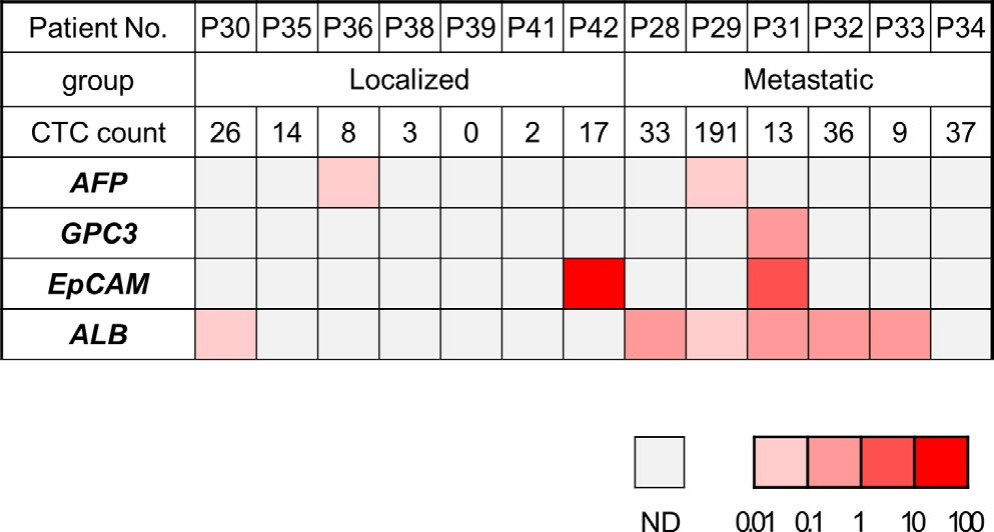
mRNA detection from CTCs was shown using a heat map in 7 patients with localized HCC (localized) and 6 patients with metastatic HCC (metastatic). The expression levels of liver‐specific genes at HuH7 mRNA concentration of 10^−5 ^ng/μL were used as the standard value, and CTC‐derived *AFP*, *GPC3*, *EpCAM*, and *ALB* expression levels were shown as relative ratios (0.01 to 100 times) to those of standard values. ND, not detected

To confirm the obtained cells as CTCs and to examine the molecular characteristics of the detected CTCs, we analyzed their gene expression using qPCR as with the analysis using clinical samples. To this purpose, mRNA was extracted from CTCs in 3 mL of whole blood from the patients. In 13 HCC patients, the detection rates of *AFP*, *GPC3*, *EpCAM*, and *ALB* mRNA were 15.4% (2/13), 7.7% (1/13), 15.4% (2/13) and 46.2% (6/13), respectively (Figure 5). Interestingly, the detection rate of *ALB* mRNA in the metastatic group was significantly higher (83.3%) than that in the localized group (14.3%) (*p* < 0.05). However, the serum albumin levels were not different between localized and metastatic HCCs, or between the CTC‐positive and CTC‐negative groups.

The results indicated that CTCs were included in the isolated cells because the mRNAs of four liver‐specific genes were detected in a subset of CTCs from clinical samples. Then, *ALB* mRNA was frequently detected in CTCs isolated from patients with metastatic HCC. Therefore, CTCs expressing *ALB* mRNA may serve as unique molecular marker of HCC patients holding highly metastatic potential.

## 4 DISCUSSION

The MCA system is a novel device for sensitive detection of CTCs from whole blood. As described previously, the MCA system showed a high recovery rate exceeding 90% of lung cancer cell lines such as A820, H441, and A549 spiked into 3 mL blood of healthy volunteers. In addition, researchers have detected larger numbers of CTCs using blood samples from patients with non‐small‐cell lung cancer. It has been reported that the MCA system achieved a superior detection of CTCs, probably because it was size dependent and not *EpCAM* expression dependent in tumor cells, such as the CellSearch system.^12^, ^13^ In the present study, we evaluated the clinical significance of the MCA system in patients with HCC. Firstly, we confirmed high recovery rates of HCC cell lines spiked into whole blood of healthy volunteer, and larger numbers of CTCs were detected in progressive HCC patients. Next, we developed a noninvasive method for the analysis of mRNA expression of liver‐specific genes derived from CTCs using qPCR. We focused on identification of positive CTCs, which may be involved with poor prognosis of HCC patients.

Recently, the review article, which showed the importance of noninvasive liquid biopsy for the diagnosis and prognosis of HCC was reported.^17^ Furthermore, some novel detection methods of CTCs have been developed in patients with HCC, because of low frequency of *EpCAM* expression in HCC. CTC detection rates of these EpCAM‐independent methods have been reported relatively high as 56% to 81% in HCC patients.^15^, ^18^, ^19^ In addition, in those size‐based methods, there is a possibility that some contaminated leukocytes were detected as CTC and may result to false positive detection of CTCs. In this study, contaminated leukocytes were tried to be excluded using immunofluorescence study. However, a small number of probable CTCs with immunofluorescent intensities that were positive for cytokeratin and DAPI, and negative for CD45 were captured on the filter even in the patients without any cancers. We considered those cells as contaminated leukocytes that were unsuccessfully excluded because of their relatively large size and non‐specific immunofluorescent reaction. Therefore, we performed ROC analysis to determine the positive cut‐off of CTC numbers, which indicates the presence of actual tumor cells circulating in blood of patients with HCC. As a result, those cut‐off numbers were defined as 10 or more, and it was significantly effective for predicting poor prognosis. It was considered that the ROC analysis may be necessary in CTC studies using the MCA system. In patients with metastatic HCC, CTC‐positive and CTC‐negative individuals had no differences in the survival rates, but we considered that survival rate was related to the number of CTCs detected even in those patients. This was because in patients with metastatic HCC, patients with 100 or more CTCs had a significantly worse prognosis than those with less than 100 CTCs. Furthermore, we investigated factors that may influence the prognostic performance of CTC numbers. In general, the prognosis of patients with HCC is strongly influenced by portal vein invasion.^20^ In this study, severe portal vein invasion was observed in all three patients with 100 or more CTCs. Therefore, this may influence the prognostic performance of CTC numbers in patients with metastatic HCC.

Some conflicting results of mRNA expression analysis need to be discussed. As shown in Figure 5, one of four mRNA expressions were detected in two patients with less than 10 CTCs. It is thought that examining some selected mRNA expressions have potential to identify the definitive presence of CTCs even below the cut‐off number in the MCA system, and that seemed to be an advantage in investigating the mRNA expressions. On the other hand, as there were two patients with 10 or more CTCs and all mRNA expressions being negative, it was suggested that a certain percentage of CTCs that did not express any of the liver‐specific mRNA we measured were existed. In previous report, Ogle LF *et al*. demonstrated in their analysis of CTCs from patients with HCC that the detection rates of *AFP*, *GPC3*, and *EpCAM* were 20%, 12.5%, and 18%, respectively, while 28% were negative for all.^19^ Interestingly, those rates including the rate of all negative patients, showed similar results in our present study. This indicates that CTCs without liver‐specific mRNA expression may be present when analyzed for a limited number of genes. This implies the significance and importance of comprehensive analysis of gene expression using a CTC such as a broad analysis by single‐cell RNA‐Seq. We performed a quality check using the residual mRNA sample to check whether the RNA sample was suitable for comprehensive analyses such as next generation sequencing (NGS). Because of the high quality of Median DV200 at 90%, it may be possible to perform a comprehensive NGS analysis in the future using these samples.

Of all four liver‐specific genes in this study, detection rate of *ALB* mRNA was the highest by qPCR analysis in patients with HCC. This result may be due to universal expression of *ALB* mRNA in CTCs compared to other relatively specific tumor markers such as *AFP*, *GPC3*, and *EpCAM*. Therefore, in the current study, detection of *ALB* mRNA is thought to be useful for the sensitive identification of CTCs. In previous study, Kar S *et al*. similarly detected *ALB* mRNA in peripheral blood in patients with advanced‐stage HCC and described that it may predict the clinical risk of tumor recurrence after surgical resection.^21^ Determining the mRNA expression for *AFP*, *GPC3*, and *EpCAM* also helps identify CTCs from patients with HCC, because they are specifically expressed in HCC and absent in normal blood cells. GPC3 is a well‐known tumor marker in HCC^15^ and accurately predicts the recurrence after curative resection of early‐stage HCC.^22^ Furthermore, GPC3 might be a target for cancer immunotherapy in patients with HCC.^23^ EpCAM is a biomarker for cancer stem cells in HCC, and overexpression of EpCAM is associated with poor prognosis in patients with HCC after curative liver resection.^24^, ^25^, ^26^, ^27^ Thus, analysis of specific and critical mRNA expression in CTCs should help characterize HCC and select therapeutic strategies, even with low positive rates of mRNA expression.

In summary, we have developed a novel strategy to analyze CTCs and mRNA expression of the CTCs in patients with HCC. Identification of positive CTCs or *ALB* mRNA expression can help predict clinical outcome of patients with HCC. The limitation of this study was its examination in a small sample of patients. Thus, larger cohorts of patients with HCC should be examined to evaluate the suitability of the MCA system for CTC enumeration and tumor characterization. Furthermore, in this study, the tumor origin of the liver‐specific mRNA, such as *AFP*, *ALB*, *GPC3* and *EpCAM* were not confirmed in the clinical samples, because the MCA system was specifically programmed for CTC detection and identification, and only the three antibodies including CK antibody, CD45 antibody, and DAPI provided by Hitachi Chemical were available in this method. Thus, it was not possible to perform additional immunofluorescent study using other antibodies. This may be one of significant limitations to the MCA system. As an advantage of this strategy, CTC detection at each stage of HCC is repeatable, because appropriate blood samples are easily obtained, and the intervention does not cause serious side effects and can be performed quickly in daily routine. Such monitoring for rare cancer cells in the blood holds considerable promise for the early detection in carcinogenesis and metastasis of cancer. Thus, clinical application of the MCA system may provide important information, especially in the field of precision medicine.

## CONFLICT OF INTEREST

Katsuya Endo and Masayuki Higuchi are employees of Hitachi Chemical Co., Ltd. The other authors have no conflict of interest.

## AUTHORS’ CONTRIBUTIONS

K. T. designed and performed the experiments, analyzed data, and wrote the paper. K. O., T. N., T. N., and H. M. helped perform the experiments and analyze the data. M. O., T. N., and K. H. helped write the paper. K.E. and M.H. provided technical information about the microcavity array system. Y.N. designed the study and guided the research presented.

## Data Availability

Research data not shared.
